# A methodology for elucidating regulatory mechanisms leading to changes in lipid profiles

**DOI:** 10.1007/s11306-017-1214-y

**Published:** 2017-05-29

**Authors:** Ferran Casbas Pinto, Srinivarao Ravipati, David A. Barrett, T. Charles Hodgman

**Affiliations:** 1 School of Biosciences, University of Nottingham, Sutton Bonington, LE12 5RD UK; 2 Centre for Analytical Bioscience, School of Pharmacy, University of Nottingham, Nottingham, NG7 2RD UK

## Abstract

**Introduction:**

It is difficult to elucidate the metabolic and regulatory factors causing lipidome perturbations.

**Objectives:**

This work simplifies this process.

**Methods:**

A method has been developed to query an online holistic lipid metabolic network (of 7923 metabolites) to extract the pathways that connect the input list of lipids.

**Results:**

The output enables pathway visualisation and the querying of other databases to identify potential regulators. When used to a study a plasma lipidome dataset of polycystic ovary syndrome, 14 enzymes were identified, of which 3 are linked to ELAVL1—an mRNA stabiliser.

**Conclusion:**

This method provides a simplified approach to identifying potential regulators causing lipid-profile perturbations.

**Electronic supplementary material:**

The online version of this article (doi:10.1007/s11306-017-1214-y) contains supplementary material, which is available to authorized users.

## Introduction

Recent advances in analytical methods, especially mass spectrometry, have shown that complex lipid profiles can be influenced by many factors and have the potential to improve our understanding of the pathophysiology underlying disease processes (references listed in Supplementary Information Section 2). However, the latter task is made difficult as a result of the complex chemical nature of lipids and their ubiquitous presence in multiple roles in biological tissues. In addition, the identification of many lipid molecular species is complicated by the high number of structural isomers corresponding to a given lipid mass. Although some low-throughput mass spectrometry techniques lead to precisely defined molecular structures (Nguyen et al. 2015), there is still more technical development needed before this can be applied in a routine way to global lipidomics. Pathways of lipid metabolism are both complex and often not fully understood: some reactions are not well characterized; some metabolites are not endogenously synthesised but enter through the diet (Holmes et al. 2008); and many enzymes have low substrate specificity, allowing them to accept a huge range of fatty acids and alcohols as substrates (Gibellini and Smith 2010). In addition, most biological and clinical scientists refer to lipids by common and often inconsistent names, rather than a systematic naming scheme, which can lead to confusion. These factors together make it difficult to automate the construction and analysis of metabolic pathways and networks, and a basic requirement for progress is to define a mechanism for resolving synonyms and one-to-many relationships in lipid identities (Horne et al. 2004).

Most recent lipidomics papers conclude their study with a list of the lipids that were found to be significantly different between two groups, for example between diseased and healthy tissue or between treated and control samples. However, there is a small number of examples that go one step further. For example, one can create and test a predictive model (Floegel et al. 2013), apply advanced statistics to classify profiles into high or low risk of suffering a disease (Rhee et al. 2011), manually construct a network between known disease-associated genes, signal-transduction pathways and the perturbed levels of specific lipids (Wang-Sattler et al. 2012), or colour-code pathway or network maps from databases such as the Kyoto Encyclopedia of Genes and Genomes (KEGG) (Cottret et al. 2010; Titz et al. 2015).

In this work, we propose a method of systematically converting lists of perturbed lipids to integrate them with metabolic network which permits visualisation of the affected pathways. This process involves four steps, the first two involving the construction of a network (that links enzymes and hence genes to reactions between specific lipids) and a thesaurus (linking generic lipid identifiers to a list of possible specific metabolites). The second two steps involve the implementation of algorithms to generate the subset of the total network that corresponds to the input profile of perturbed lipids, and to rank enzyme nodes in terms of their significance to the perturbation. As a proof of concept, we apply this method to a published lipidomics dataset .

## Methods

### Data sources

Source data for creating the holistic human lipidomic network included LIPID MAPS (Sud et al. 2007), KEGG (Kanehisa 1996), HMDB (Wishart et al. 2013) and other papers cited in Supplementary Information Section 3.

### Network construction

The lipid metabolic network was created using Python scripts applied to data from the above sources, plus manual curation, and has been stored in a mySQL database. All scripts can be obtained from https://github.com/Ferran-Casbas/Lipid-Pathway. The network contains a metabolite node for each specific lipid, but does not distinguish between the fatty-acyl groups at the sn-1 and sn-2 chain positions to limit the network size and complexity and because analytical resolution of sn-1/sn-2 sidechains is rarely achieved across the whole lipidome. Other nodes may refer to individual reactions, and the enzymes (specified by their Human Genome Identifier) catalysing them. Further details are in the Supplementary Information Section 4.

### Subset generation

**Fig. 1:**
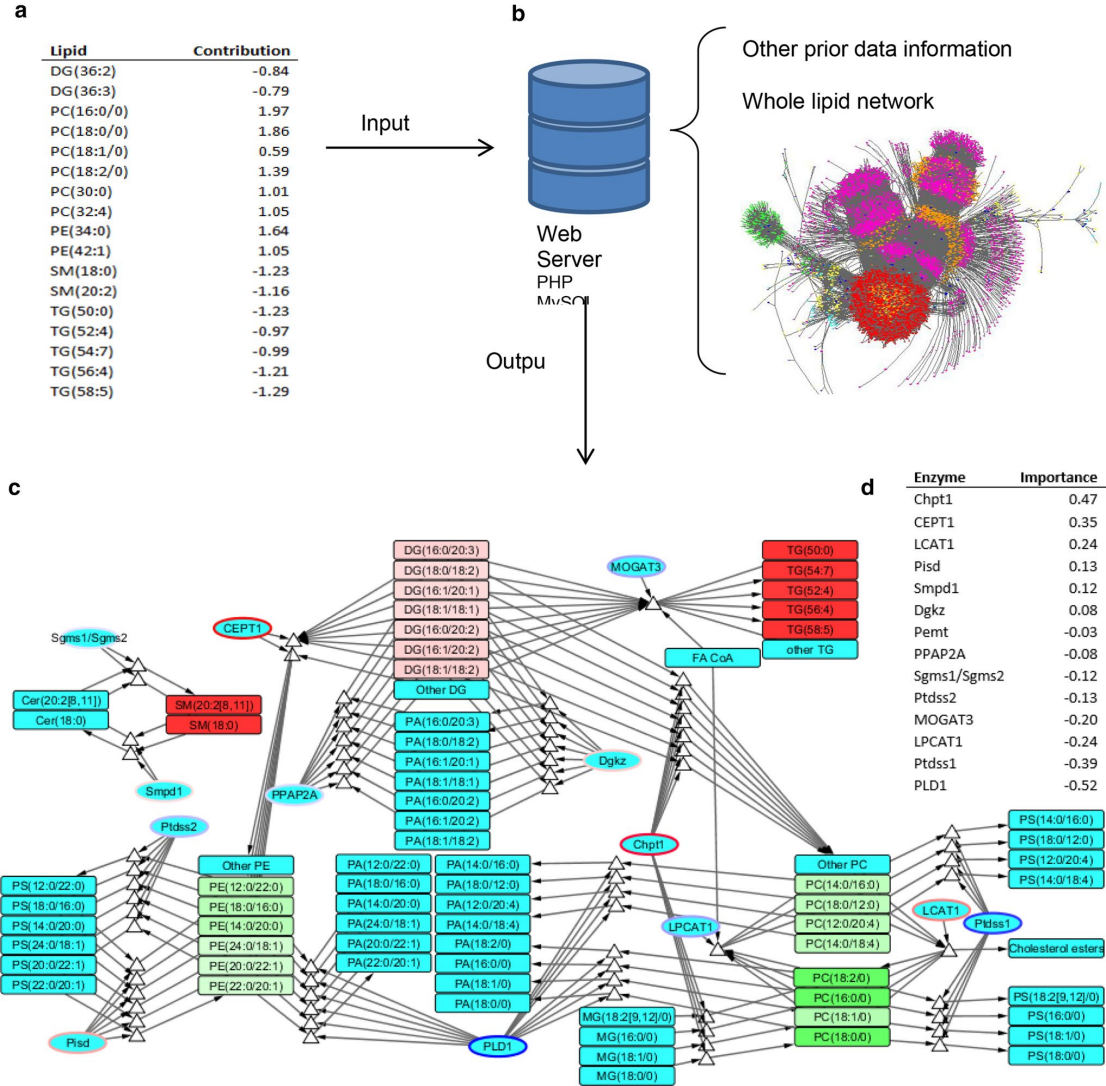
The Lipid network method. **a** Input list of lipids with OPLS-DA model scores. **b** Depicts the web server that contains scripts and the lipidomic network, in which node colours *green, dark blue, orange, red* and *white* respectively correspond to glycerophospholipids, sphingolipids, diglycerides, enzymes and specific reactions. **c** Derived subset of lipid metabolism linking the input perturbed metabolites; metabolite, reaction and enzyme nodes are respectively represented by *rectangles, triangles* and *ellipses*; input metabolite nodes are respectively coloured a *brighter shade* or *green* or *red* depending on the extent to which their levels have been elevated or decreased, or *blue* if they correspond to nodes introduced during the subset retrieval; the borders of enzyme nodes are *shaded blue* or *red* respectively to depict the extent of positive or negative Relative Importance Score (the sign corresponding to the increase or decrease in enzyme activity). **d** The list of retrieved enzymes ranked by Relative Importance

The whole process is depicted in Fig. 1. The input used is a list of lipids whose concentrations are significantly higher or lower between two groups, determined for example after univariate or multivariate data analysis of a lipidomics dataset and the structural confirmation of the chemical identities of the changed lipids. Lipid names should conform to the standard in LIPID MAPS (http://www.lipidmaps.org/data/classification/lipid_cns.html#N). The preferred level of identification is to include the lipid class and the numbers of carbons and double bonds in each side chain. For example, this approach can include ambiguously identified molecules such as DG(36:2) which indicates a diglyceride with two side chains totalling 36 carbons with 2 double bonds. The fold change or significance score for the metabolites (often the VIP scores from Partial Least Squares analysis) can also be input and used for metabolic prioritisation. Alternatively, +1 and −1 can be used to imply the direction of change in lipid level between one group and another.

PHP scripts then use the list to produce a subgraph (sub-network). The first phase of the analysis works through the list to identify specific, ambiguous and unrecognisable terms. The software reports on this and recommends what might be done to clarify the unrecognised terms. Once the lipids of interest have been adequately associated with network nodes, a sub-network is generated by selecting from the lipidomic network these nodes and their two nearest neighbours, so that the reactions and enzyme/genes linking these lipids are also included. If the input lipids have fold change or significance scores, the output sub-network contains *Relative Importance* scores, which indicate how much influence the enzyme nodes have on producing the observed lipid-profile perturbation in light of the network topology. This can be particularly valuable when larger sub-networks are returned. More information on this is in Supplementary Information Section 6.

The above scripts are available through a web interface, which can be found at: http://lipidnetwork.nottingham.ac.uk/. The web tool outputs two files which can be imported into a network visualization software, such as Cytoscape (Doerks et al. 2002), to see the metabolic pathways involved in the profile changes. Another option is to open the node-info file in a spreadsheet to retrieve the list of enzymes, which can then be used as a query to other network tools, such as the BioGRID (Stark et al. 2006) or genemania, to identify potential regulators.

## Results and discussion

This work describes a novel method for identifying enzymes and potential regulators related to measured changes in the lipid profile obtained from mass spectrometry analysis. The method includes the development of a holistic lipidomic network that comprises 127 enzymes, 13,934 reactions and 7561 metabolites. The latter are from 16 lipid subclasses: 3124 glycerolipids, 3575 glycerophospholipids, 290 sphingolipids, 126 fatty acyls (including eicosansoids, fatty amides, esters and their conjugates), and 446 miscellaneous lipids. An image of the complete network can be seen in Supplementary Fig. 1, and further details of the network’s properties are described in Supplementary Tables 1–3. The Supplementary Information Section 9 outlines extensions to this method that can be carried out by suitably experienced bioinformaticians.

To show the power and utility of the method, it has been applied to a published dataset that contains lipid profile for women with polycystic ovary syndrome (PCOS) compared with matched controls (2). The contribution score of the lipids found in significantly different concentration was assigned from an OPLS-DA analysis (see Fig. 1). The output .sif and node-attributes files were imported into Cytoscape and the nodes manually stacked to aid visual interpretation of the network. This allows us to view the enzymes that have a higher impact on the profile.

In this example, 14 enzymes have been retrieved falling into two separate components, with the highest in absolute value of Relative Importance scores being for PLD1 (a phosphatidylcholine-specific phospholipase) and Chpt1 (a choline phosphotransferase). This suggests that the changes in plasma lipid profile associated with PCOS may be dominated by a reduction in Chpt1 activity and/or an increase in PLD1 activity. The former is responsible for changes in DG and PC, and PLD1 is responsible for changes in PE and PC. Since there is equilibrium between the PA and DG species, a simple hydrolysis would convert one into the other, the perturbation of Pld1 could by itself explain the majority of observed changes.

The list of enzymes provided by this metabolic insight can be used to query other biomolecular network tools to identify potential common regulators of these lipids. 11 of them were present in BIOGRID, 4 linked by ELAVL1 and 2 by ILF3, which both protect target mRNAs from degradation (see Supplementary Fig. S7). Querying GeneMania revealed consistent patterns for regulation of Chpt1 and Pisd by microRNAs recognising the target sequence TGCTTGCT, and Dgkz, Ppap2a and Smpd1 by the transcription factors MAZ, NFAT and STAT5B (Supplementary Fig. S8). Unfortunately, both regulators have an enzyme whose activity goes in the opposite direction to the rest, so it is difficult to assert any strong hypotheses at this stage.

## Conclusions

To conclude, the methodology presented here provides a way to move from tables of perturbed lipid levels to a visualization of the metabolic network that could cause these changes and thence to potential regulators. It also provides a metric (*Importance*) to rank the positive or negative contribution of each enzyme to produce the changes seen.

## Electronic supplementary material

Below is the link to the electronic supplementary material.

Supplementary material 1 (DOCX 6061 KB)

Supplementary material 2 (TIFF 3845 KB)
