# Supplementary material for: A methodology for elucidating regulatory mechanisms leading to changes in lipid profiles
Source: Metabolomics. 2017 May 29;13(7):81. doi: 10.1007/s11306-017-1214-y (PMC5447331; doi:10.1007/s11306-017-1214-y)
Supplement: Supplementary file 1 — Supplementary material 1 (DOCX 6061 KB) [file 11306_2017_1214_MOESM1_ESM.docx]

A methodology for elucidating regulatory mechanisms leading to changes in lipid profiles

Ferran Casbas Pinto^1,2^, Srinivararao Ravipati^2^, David A. Barrett^2^, T. Charles Hodgman^1^*

^1^ School of Biosciences, University of Nottingham, Sutton Bonington, LE12 5RD, UK

^2^ Centre for Analytical Bioscience, University of Nottingham, Nottingham, NG7 2RD, UK

*** Corresponding author**

1. Abbreviations

CEPT1, Choline/Ethanolamine Phosphotransferase 1; CHPT1, Choline Phosphotransferase; DG, Diglyceride; HPLC, High-performance liquid chromatography; LPCAT, Lysophosphatidylcholine Acyltransferase; MG, Monoglyceride; MOGAT3, Monoacylglycerol O-Acyltransferase 3; MS, mass spectrometry; OPLS-DA, Orthogonal partial least squares discriminant analysis; PCOS, Polycystic ovary syndrome; PHP, Hypertext Pre-processor; Pld1, Phospholipase D1, Phosphatidylcholine-Specific; PLS, Partial least squares; Sgms1, Sphingomyelin Synthase 1; Smpd1, Sphingomyelin phosphodiesterase .

2. Lipidomic references

Assfalg, M., I. Bertini, D. Colangiuli, C. Luchinat, H. Schafer, B. Schutz, et al. (2008). Evidence of different metabolic phenotypes in humans. Proc Natl Acad Sci U S A 105, 1420-4 doi:10.1073/pnas.0705685105

Cordeiro, F. B., T. R. Cataldi, K. J. Perkel, L. do Vale Teixeira da Costa, R. C. Rochetti, J. Stevanato, et al. (2015). Lipidomics analysis of follicular fluid by ESI-MS reveals potential biomarkers for ovarian endometriosis. Journal of Assisted Reproduction and Genetics, doi:10.1007/s10815-015-0592-1

Dallmann, R., A. U. Viola, L. Tarokh, C. Cajochen, S. A. Brown (2012). The human circadian metabolome. Proc Natl Acad Sci U S A 109, 2625-9 doi:10.1073/pnas.1114410109

Haoula, Z., S. Ravipati, D. Stekel, C. Ortori, C. Hodgman, C. Daykin, et al. (2015). Lipidomic analysis of plasma samples from women with polycystic ovary syndrome. Metabolomics 11, 657-666 doi:10.1007/s11306-014-0726-y

Hashmi, S., Y. Wang, D. S. Suman, R. S. Parhar, K. Collison, W. Conca, et al. (2015). Human cancer: is it linked to dysfunctional lipid metabolism? Biochim Biophys Acta 1850, 352-64 doi:10.1016/j.bbagen.2014.11.004

Holmes, E., R. L. Loo, J. Stamler, M. Bictash, I. K. Yap, Q. Chan, et al. (2008). Human metabolic phenotype diversity and its association with diet and blood pressure. Nature 453, 396-400 doi:10.1038/nature06882

Illig, T., C. Gieger, G. Zhai, W. Romisch-Margl, R. Wang-Sattler, C. Prehn, et al. (2010). A genome-wide perspective of genetic variation in human metabolism. Nat Genet 42, 137-41 doi:10.1038/ng.507

Kenny, L. C., D. I. Broadhurst, W. Dunn, M. Brown, R. A. North, L. McCowan, et al. (2010). Robust early pregnancy prediction of later preeclampsia using metabolomic biomarkers. Hypertension 56, 741-9 doi:10.1161/HYPERTENSIONAHA.110.157297

Krug, S., G. Kastenmuller, F. Stuckler, M. J. Rist, T. Skurk, M. Sailer, et al. (2012). The dynamic range of the human metabolome revealed by challenges. FASEB J 26, 2607-19 doi:10.1096/fj.11-198093

Liu, Q., J. Zhang (2014). Lipid metabolism in Alzheimer's disease. Neurosci Bull 30, 331-45 doi:10.1007/s12264-013-1410-3

Oresic, M., S. Simell, M. Sysi-Aho, K. Nanto-Salonen, T. Seppanen-Laakso, V. Parikka, et al. (2008). Dysregulation of lipid and amino acid metabolism precedes islet autoimmunity in children who later progress to type 1 diabetes. J Exp Med 205, 2975-84 doi:10.1084/jem.20081800

Shaham, O., R. Wei, T. J. Wang, C. Ricciardi, G. D. Lewis, R. S. Vasan, et al. (2008). Metabolic profiling of the human response to a glucose challenge reveals distinct axes of insulin sensitivity. Mol Syst Biol 4, 214 doi:10.1038/msb.2008.50

Xu, J., A. M. Casas-Ferreira, Y. Ma, A. Sen, M. Kim, P. Proitsi, et al. (2015). Lipidomics comparing DCD and DBD liver allografts uncovers lysophospholipids elevated in recipients undergoing early allograft dysfunction. Sci Rep 5, 17737 doi:10.1038/srep17737

Yu, X. H., N. Jiang, X. L. Zheng, F. S. Cayabyab, Z. B. Tang, C. K. Tang (2014). Interleukin-17A in lipid metabolism and atherosclerosis. Clin Chim Acta 431, 33-9 doi:10.1016/j.cca.2014.01.012

3. Supplementary Input data sources

Haoula, Z., S. Ravipati, D. Stekel, C. Ortori, C. Hodgman, C. Daykin, et al. (2015). Lipidomic analysis of plasma samples from women with polycystic ovary syndrome. Metabolomics 11, 657-666 doi:10.1007/s11306-014-0726-y

Haughey, N. J., L. B. Tovar-y-Romo, V. V. Bandaru (2011). Roles for biological membranes in regulating human immunodeficiency virus replication and progress in the development of HIV therapeutics that target lipid metabolism. J Neuroimmune Pharmacol 6, 284-95 doi:10.1007/s11481-011-9274-7

Hiley, C. R., P. M. Hoi (2007). Oleamide: A Fatty Acid Amide Signaling Molecule in the Cardiovascular System? Cardiovascular Drug Reviews 25, 46-60 doi:10.1111/j.1527-3466.2007.00004.x

Moessinger, C., K. Klizaite, A. Steinhagen, J. Philippou-Massier, A. Shevchenko, M. Hoch, et al. (2014). Two different pathways of phosphatidylcholine synthesis, the Kennedy Pathway and the Lands Cycle, differentially regulate cellular triacylglycerol storage. BMC Cell Biology 15, 43

Toke, D. A., C. E. Martin (1996). Isolation and Characterization of a Gene Affecting Fatty Acid Elongation in Saccharomyces cerevisiae. Journal of Biological Chemistry 271, 18413-18422 doi:10.1074/jbc.271.31.18413

Wishart, D. S., C. Knox, A. C. Guo, R. Eisner, N. Young, B. Gautam, et al. (2009). HMDB: a knowledgebase for the human metabolome. Nucleic Acids Res 37, D603-10 doi:10.1093/nar/gkn810

Wishart, D. S., D. Tzur, C. Knox, R. Eisner, A. C. Guo, N. Young, et al. (2007). HMDB: the Human Metabolome Database. Nucleic Acids Res 35, D521-6 doi:10.1093/nar/gkl923

4. Manual curation

Before using the python script to generate the global lipid network a network skeleton is required. A skeleton network consisting of the generic reactions associated with enzymes of lipid metabolism was obtained from LipidMaps in conjunction with publications of lipid metabolism. The python script, named [Network-Multiplicator.py](https://github.com/Ferran-Casbas/Lipid-Network/blob/master/Network-Multiplicator.py), was then used to generate the global lipid network, by creating reactions specific to all the alternative substrate molecules, and associating these reaction nodes with the enzyme catalysing them. Two reactions have been excluded from the lipidomic network: Lysophosphatidylcholine Acyltransferase 1 (LPCAT1) and lecithin-cholesterol acyltransferase (LCAT1), which are both involved in PC-LysoPC conversion. This is because they add so many links that a large number of irrelevant PC nodes are returned. To compensate for this, the subset-extraction algorithm adds these reactions back in when any LysoPC and PC is found in the subnetwork generated. In this way, the program still reveals the potential role of these enzymes in producing the observed lipidome perturbations.

5. Lipidomic network properties

Table S1. Number of lipids and families in the total network

| **Families** | **Subfamily** | **Count** |
| --- | --- | --- |
| Fatty acyls **(126)** | Fatty amides | 44 |
|  | Eicosanoids | 36 |
|  | Fatty esters | 23 |
|  | Fatty acid conjugates | 23 |
| Glycerolipids **(3124)** | MG | 44 |
|  | DG | 1056 |
|  | TG | 2024 |
| Glycerophospholipids **(3575)** | PC | 825 |
|  | PS | 275 |
|  | PA | 825 |
|  | PE | 825 |
|  | PI | 275 |
|  | PG | 550 |
| Sphingolipids **(290)** | Ceramides | 88 |
|  | SM | 44 |
|  | Other sphingolipids | 158 |
| Others lipid metabolites **(446)** |  | 446 |
| **Total** |  | **7561** |


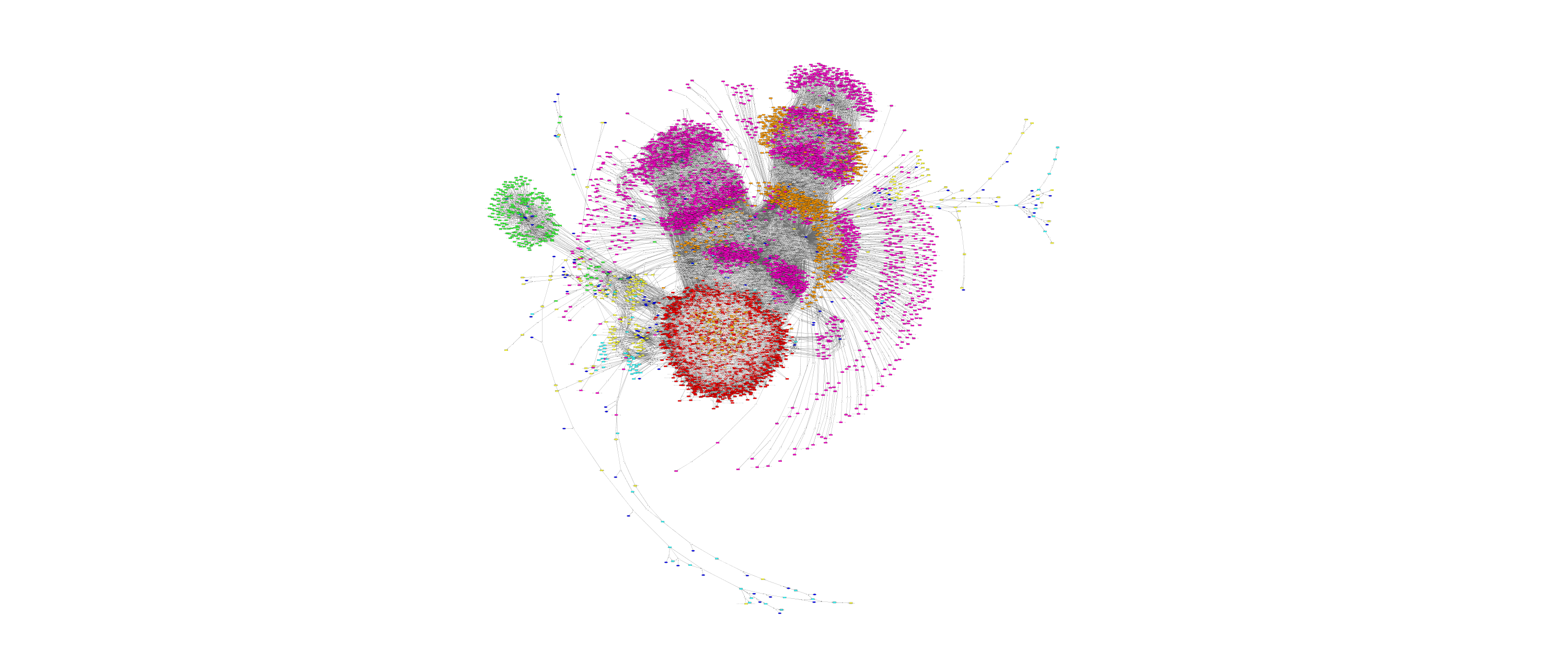


Fatty acyls

Glycerolipids

Glycerophospholipids

Sphingolipids

Other lipids

Enzymes

Triglycerides

FigS1. The whole network of human lipid metabolism.

The box denotes the colour scheme of nodes, excluding reaction nodes coloured white and hence not visible. The links between enzyme, lipid and reaction nodes are coloured grey. The nodes and edges have been positioned using the “organic layout” algorithm within Cytoscape. There is a clear separation of sphingolipids (in green) from other lipid classes. The mono- and di-glycerides (orange) form precursors for various phospholipids and other lipid types.

Tables S2 and S3 respectively present the top lipid and enzyme nodes ranked by vertex degree, along with associated network properties: stress (the number of shortest paths between all nodes that cross the specified node), degree (the number of adjacent neighbours of a node), betweenness (the node stress divided by the number of alternative paths of the same length). The final column states their ranking according to stress.

Table S2. Lipid node metrics

| **Name** | **Stress** | **Degree** | **Betweenness** | **Rank Stress** |
| --- | --- | --- | --- | --- |
| 5Z,8Z,11Z,14Z-eicosatetranoyl CoA | 1.93E+10 | 363 | 0.022 | 23 |
| Hexadecanoyl CoA | 1.32E+10 | 363 | 0.013 | 30 |
| Octadecanoyl CoA | 1.32E+10 | 363 | 0.013 | 29 |
| Oleoyl CoA | 1.29E+10 | 363 | 0.012 | 33 |
| Docosanoyl CoA | 1.30E+10 | 362 | 0.013 | 32 |
| 7Z,10Z,13Z,16Z-Docosatetraenoyl CoA | 1.28E+10 | 362 | 0.012 | 34 |
| 13Z-Docosenoyl CoA | 1.28E+10 | 362 | 0.012 | 35 |
| 8Z,11Z-eicosadienoyl CoA | 1.25E+10 | 362 | 0.012 | 39 |
| Linoleoyl CoA | 1.28E+10 | 361 | 0.015 | 36 |
| 4Z,7Z,10Z,13Z,16Z,19Z-Docosahexaenoyl CoA | 1.19E+10 | 361 | 0.012 | 45 |

Table S3. Enzyme node metrics

| **Name** | **Stress** | **Degree** | **Betweenness** | **Rank Stress** |
| --- | --- | --- | --- | --- |
| MOGAT3 | 8.79E+10 | 7200 | 0.379 | 2 |
| CEPT1 | 2.68E+11 | 1500 | 0.278 | 1 |
| Pemt | 2.9E+10 | 924 | 0.055 | 19 |
| PPAP2A | 2.24E+09 | 600 | 0.036 | 92 |
| PLD1 | 5.97E+10 | 600 | 0.026 | 8 |
| Agpat4/Agpat6 | 5.32E+09 | 600 | 0.019 | 61 |
| UG7 | 1.23E+09 | 600 | 0.008 | 242 |
| Cds1 | 6.54E+09 | 324 | 0.025 | 57 |
| Cdipt | 1.64E+09 | 324 | 0.010 | 116 |
| Chpt1 | 1.65E+09 | 324 | 0.006 | 115 |

The majority of the top-ranked metabolites in Table S2 are associated with the later stages of fatty-acyl chain elongation and formation of membrane lipids. This is reasonable since these are central to lipid metabolism. Their stress rankings are high but not remarkably so. In the case of the enzymes, however, the vertex degrees and betweenness are much higher, most especially with regard to MOGAT3 (involved in triglyceride formation) and CEPT1 (PE formation from diglycerides). Given that these enzymes can carry out a large number of different reactions, it is possible that they will appear more often in perturbed lipid profiles and this should be taken into consideration when drawing conclusions about the processes producing the observed profile changes.

6. Scoring

A metric has been defined to indicate the extent to which enzymes involved in lipid metabolism are contributing to the observed differences in lipid profiles. This contribution, i.e. *Importance*, can be obtained from the quantitative figures from the initial profile analysis. However, simply adding up the values of every lipid binding to an enzyme will result in values that vary between experiments simply because the number of input lipids is different. Hence, *Relative Importance*, which divides these input values by the number of metabolites, provides a metric that is independent of the number of molecules obtained from the lipidomic analysis. Relative Importance values are usually <1. An enzyme would only acquire a score of 1 if it explained all the perturbed lipid levels, i.e. no other enzymes were involved.

The input is a list of lipid metabolites with their score (fold change or some other statistical value). If the user only knows the direction of a perturbed lipid (i.e. the level increases or decreases), then values of +1 and -1 can be used. The number of metabolites in the list is determined, and the lipid values divided by this number to give relative values. Then, for each metabolite, the global network is queried to find the reaction nodes to which it is linked and then the enzyme(s) linked to those reactions.  A set of enzymes with their reaction nodes are stored in the dictionary. Figure S2 shows the way to calculate Relative Importance scores.


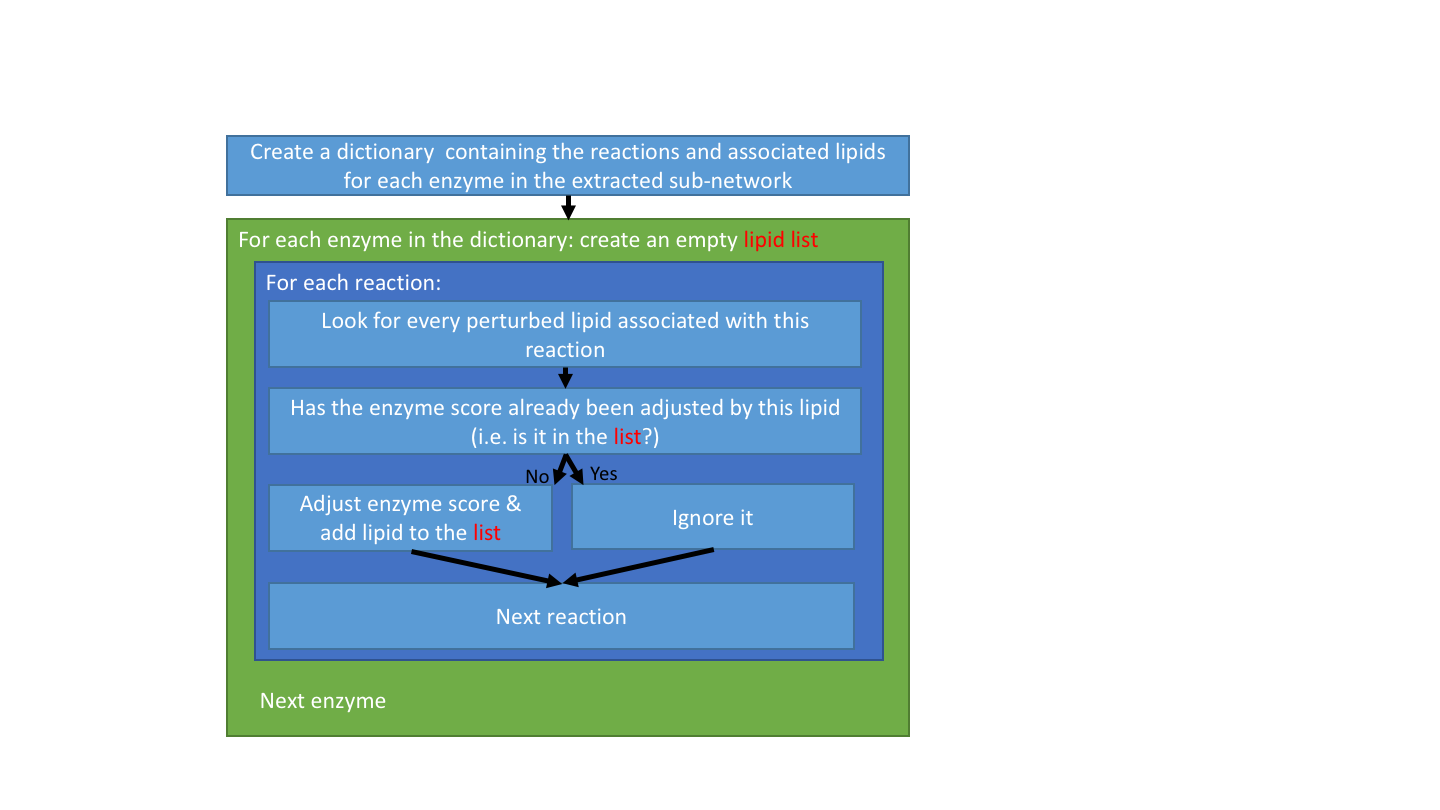


FigS2: The Relative Importance scoring function. The steps are indicated in white text with arrows indicating the order in which they are carried out. The lipid list is denoted by red text, and the nested loops for each enzyme and reaction are respectively shown by green and blue boxes.

For the first enzyme in the dictionary, an empty lipid list is created. For the first reaction, its substrate molecules are taken in turn. If the metabolite is already in the lipid list, the next substrate molecule is considered, otherwise the lipid score is subtracted from the enzyme score (because a higher substrate score indicates less enzyme activity) and the metabolite name is added to the lipid list. Next, the reaction product molecules are taken in turn. If the metabolite is already in the lipid list, the next product molecule is considered, otherwise the lipid score is added to the enzyme score (because a higher product score indicates more enzyme activity) and the metabolite name is added to the lipid list.

At this point the next reaction is taken, and the above steps repeated. Once all the reactions for the specified enzyme have been processed, the enzyme name is stored and its Relative Importance score in one array, and the enzyme, reaction and metabolite IDs as nodes and their associated edges (for laying out subnetwork) in a separate array. The above steps are repeated for each of the remaining enzymes in the dictionary. Finally, the output consists of the content of the latter two arrays. Enzymes with elevated concentrations of substrates and products will have low Relative Importance, as it does not seem to be the responsible of observed metabolite increases on both sides of its reaction. They can be considered as intermediates in a pathway, rather than a controlling step.

7. Worked Example

A list of lipids with significantly different concentrations in patients with Poly-Cystic Ovary Syndrome (PCOS) vs controls has been published (1), see Table S4. They are divided into those that are perfectly identified (where LipidMaps IDs have been assigned) and imperfectly identified (where the word General has been inserted). The Contribution values are the output from the OPLS-DA model used to identify the perturbed metabolites. In this instance, negative numbers refer to elevated levels in the patients. These data are input into the Lipid Network Creator web form at <http://lipidnetwork.nottingham.ac.uk/> as shown in Figure S3. The Scored box should be ticked, then the Create Network button is clicked. Note, SM(20:2[8,11]) is a precisely defined metabolite. Although it does not have a LipidMaps ID, it can be placed in the list with lipids with definitive identities.

Table S4. Perturbed lipid concentrations in PCOS patients

| Lipid | Lipid Maps ID | Contribution |
| --- | --- | --- |
| PC(16:0/0) | LMGP01050018 | 1.97 |
| PC(18:0/0) | LMGP01050026 | 1.86 |
| PC(18:1/0) | LMGP01050029 | 0.59 |
| PC(18:2/0) | LMGP01050035 | 1.39 |
| SM(18:0) | [LMSP03010001](http://www.lipidmaps.org/data/LMSDRecord.php?LMID=LMSP03010001) | -1.23 |
| SM(20:2[8,11]) | - | -1.16 |
| DG(36:2) | General | -0.84 |
| DG(36:3) | General | -0.79 |
| PC(30:0) | General | 1.01 |
| PC(32:4) | General | 1.05 |
| PE(34:0) | General | 1.64 |
| PE(42:1) | General | 1.05 |
| TG(50:0) | General | -1.23 |
| TG(52:4) | General | -0.97 |
| TG(54:7) | General | -0.99 |
| TG(56:4) | General | -1.21 |
| TG(58:5) | General | -1.29 |


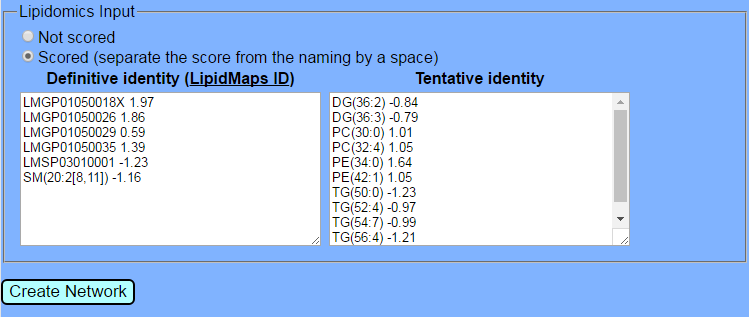
FigS3: Screenshot of the Lipid Network Creator input boxes. Because Contribution scores are available, the “Scored” tickbox has been selected. There is a typo mistake marked with a red ellipse. N.B. a single white space should separate lipid names/IDs from their scores.

The web tool returns a page containing a table of metabolites with their associated node names from the Lipid Network (see Figure S4). Unassigned lipids, shown in red text, are separated out for the user to check and amend until all the input data are in the correct format. It is possible that a lipid is not in the network, in which case we encourage the user to send an email with details, so that it might be included.


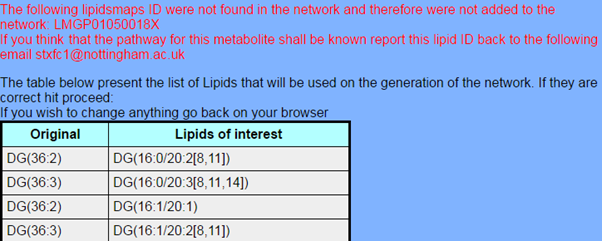


FigS4. Lipid checking page. Red text indicates that there are issues with the input names.

When all is correct, the Submit is clicked again. It may take several minutes for the results page to appear. This contains the option to download a Zip file that contains the network file (in .sif format), node attributes (as a text file), instructions for network visualisation using Cytoscape (in README.docx) (see Figure S5). These files can also be viewed directly through the web browser via the relevant hyperlinks.


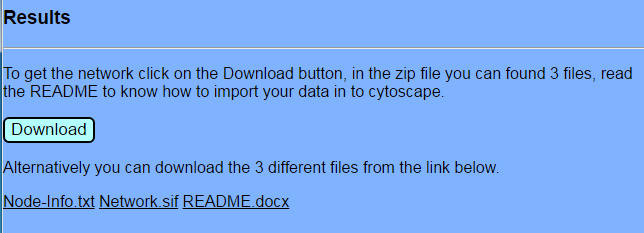


FigS5: Lipid Network Creator results page

The downloaded files should be extracted and the instructions in the Read-me file followed. This leads to a network resembling that of Figure S6.


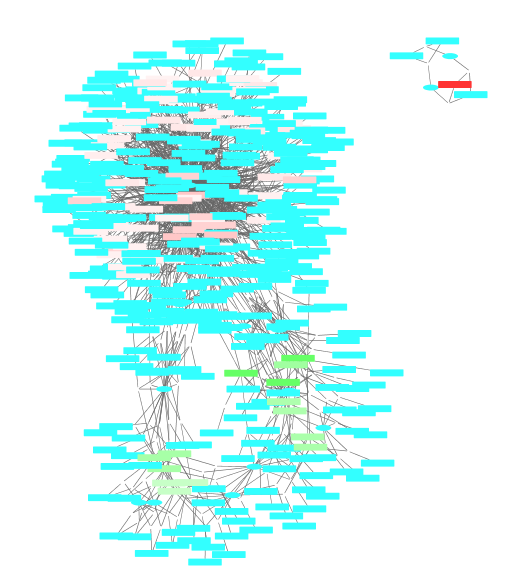


FigS6. Initial cytoscape layout. Metabolite, reaction and enzyme nodes are respectively represented by rectangles, triangles and ellipses; input metabolite nodes are respectively coloured a brighter shade or green or red depending on the extent to which their levels have been elevated or decreased, or blue if they correspond to nodes introduced during the subset retrieval. The borders of enzyme nodes are shaded blue or red respectively to depict the extent of positive or negative Importance score (the sign corresponding to the increase or decrease in enzyme activity).

Then, using the “align and distribute tool” found in the layout menu in Cytoscape, all the lipid and reaction nodes from the same family are grouped together. For example, the triglyceride nodes can be selected by typing “tg” in the search box and pressing enter. All the TG nodes are now coloured yellow. From the tool panel, click on one of the vertical stack options to align the nodes which can then be moved to one side of the network (see Figure S5). For convenience, tall stacks can be reordered into rectangular blocks of nodes for later ease of movement. This process is then repeated for other metabolite families (See Figure S6).

A B


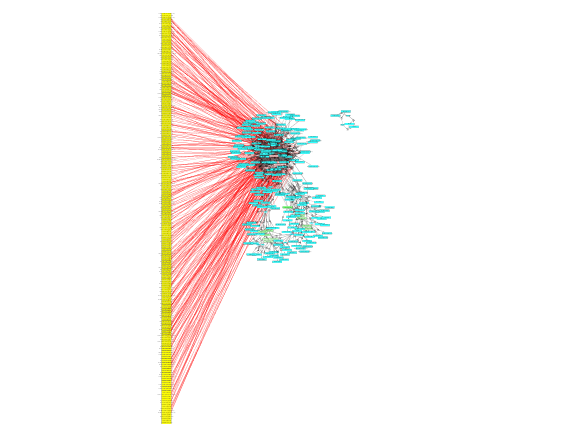

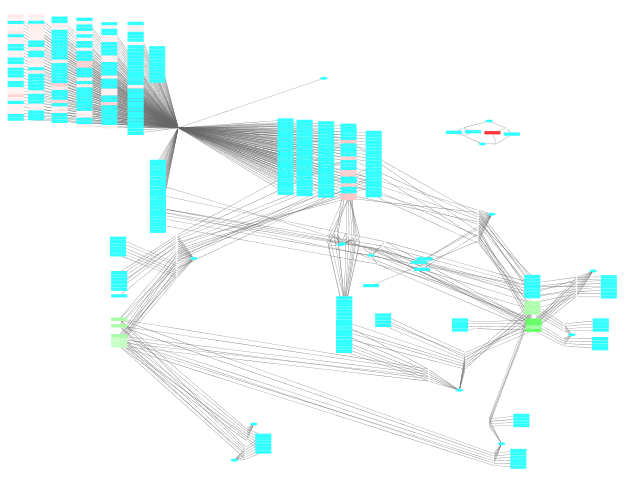


FigS7. TG stack. A. The network from Figure S6 has had the triglycerides selected, stacked and moved to the left. The TG nodes are coloured yellow and their associated edges read. Otherwise the colour scheme is the same as for Figure S4. B. The network after further stacking of metabolites.

The network now has nodes corresponding to metabolites whose concentrations are not thought to vary, and a large number of reaction nodes for each enzyme. These can be grouped together and collapsed into single nodes. From a given family of metabolite nodes, the blue nodes can be selected, then a right mouse click (or ctrl + mouse-pad click on Apple machines) provides a menu in which the nodes are first defined as a group. The same steps can then be used to collapse these nodes into a single “group node”, named, for example, “other TGs”. This node, in the table panel should then be assigned the Type “Metabolite”, and the Basic and Importance scores of 0. Sets of common reaction nodes can be grouped and collapsed in the same way. The main difference is that the Type of node should be redefined as “Reaction Node”. These steps eventually lead the network layout to something easier for the casual reader to understand (see Figure S8).


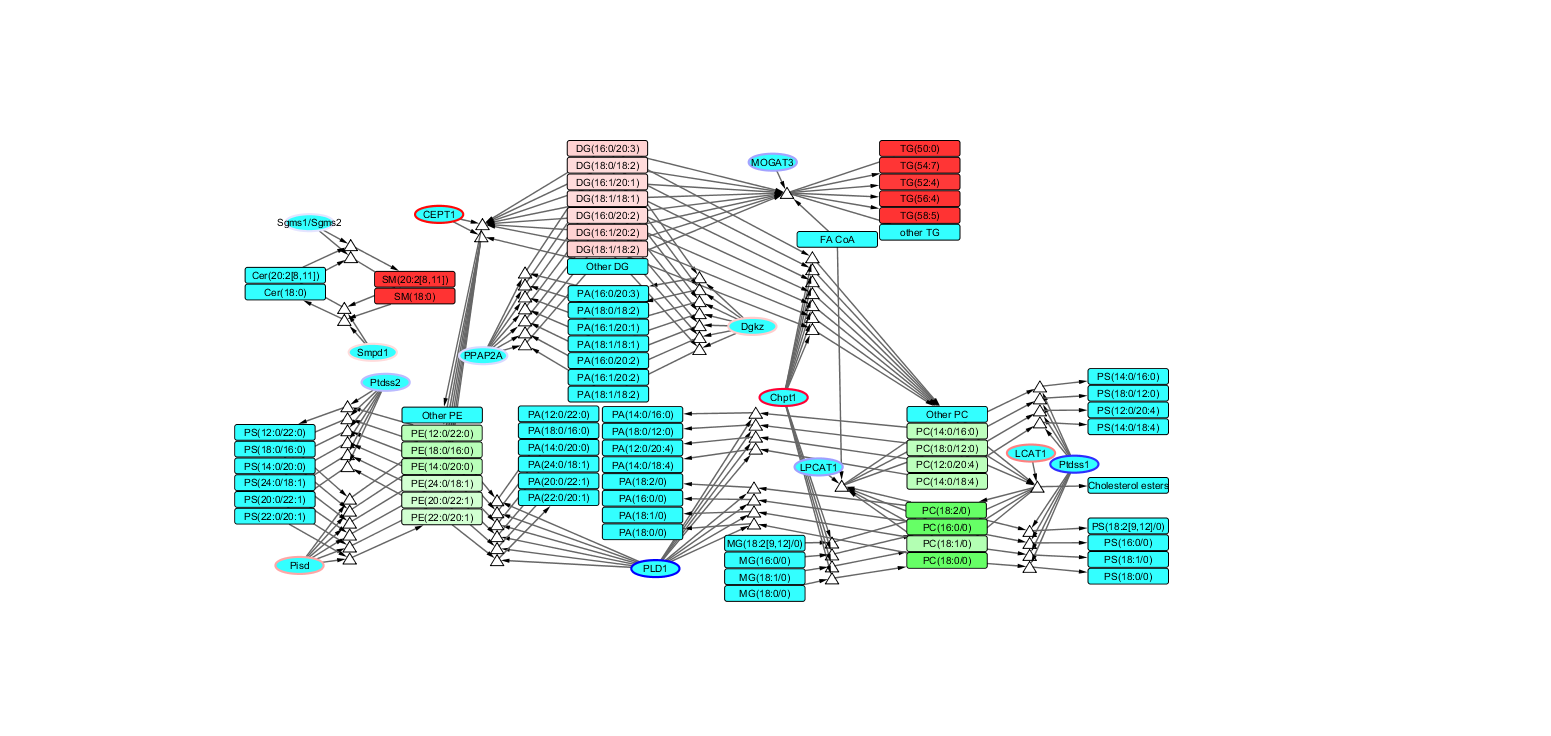


FigS8. Final network layout. Same colour patterns as S4. Also the shape of the node indicates its type, being rounded is an enzyme, square metabolite and triangles are Reaction nodes. The out colour in the enzymes indicates their importance value, meaning that red are expected under activated enzymes and blue are active enzymes.

8. Search for potential regulators

The NodeAttributes&Score file can be filtered to find the enzymes/genes of importance. These IDs can then be used to find potential common regulators, by first be pasting them into a txt file with all letters converted to upper case. This provides a set for querying interaction and regulatory databases. The BIOGRID is an excellent catalogue of protein complexes and regulatory interactions. For the readers’ convenience, a subset of BIOGRID (containing only the immediate neighbours of the lipid enzymes) has been generated. This network can be opened in Cytoscape, the nodes with Importance scores selected (using the submenu Select=>Nodes=>From ID List file…. and selecting the text file for the enzyme list), selecting first neighbours (using the submenu Select=>Nodes=>First Neighbours of Selected Nodes=>Undirected), and creating a daughter menu. Rearranging this results in Figure S9. Note, MOGAT3 is not present in the release of BIOGRID used here.


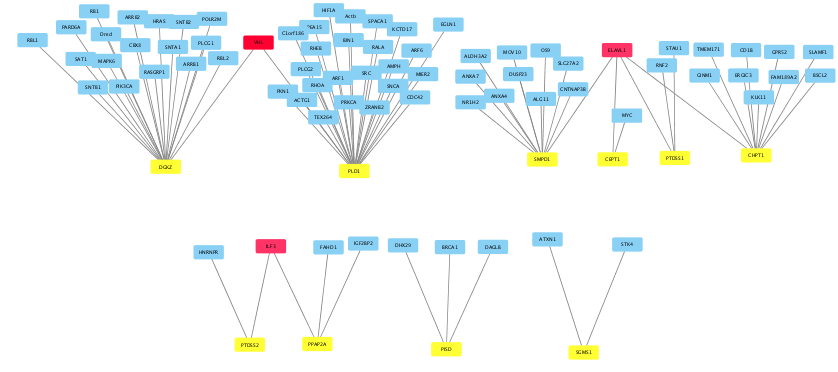


FigS9. First neighbours from the human BIOGRID. The PCOS query nodes are shown in yellow. Blue and red nodes correspond to proteins in BIOGRID which interact with 1 and >1 PCOS nodes respectively. The latter could represent co-regulators. The edges denote interactions catalogued in BIOGRID.

The figure shows that PLD1 interacts with more proteins than any other, which might be expected of an enzyme involved in many different reactions. ELAVL1 is an mRNA-stabilising protein shown to bind to the transcripts for CHPT1, CEPT1, SMPD1 and PTDSS1 (Abdelmohsen et al. 2009). It could play a role here by stabilising the transcripts for the first three enzymes whose activity goes up. Why the activity of PTDSS1 goes down remains unclear. Likewise, ILF3 is a co-regulator of PTDSS2 and PPAP2A (Havugimana et al. 2012), again at the mRNA level, but their activities also go in opposite directions. Other interaction databases can also be queried. GeneMania is one, which includes data on gene and microRNA-mediated regulation and can be accessed via Cytoscape. The gene-regulatory subset for the PCOS gene list is shown in Figure S10. This suggests possible roles for MAZ-Q6, NFAT-04 and STAT5B transcription factors, with the latter two possibly explaining the increased activity of SMPD1 and DGKZ, and DGKG, a non-catalytic sub-unit of the DGK complex. Likewise, there is a possible role for microRNA regulation by MIR15, 19, 424 or 497. A decrease in their levels could contribute to the elevated activities of CEPT1 and PISD.

**
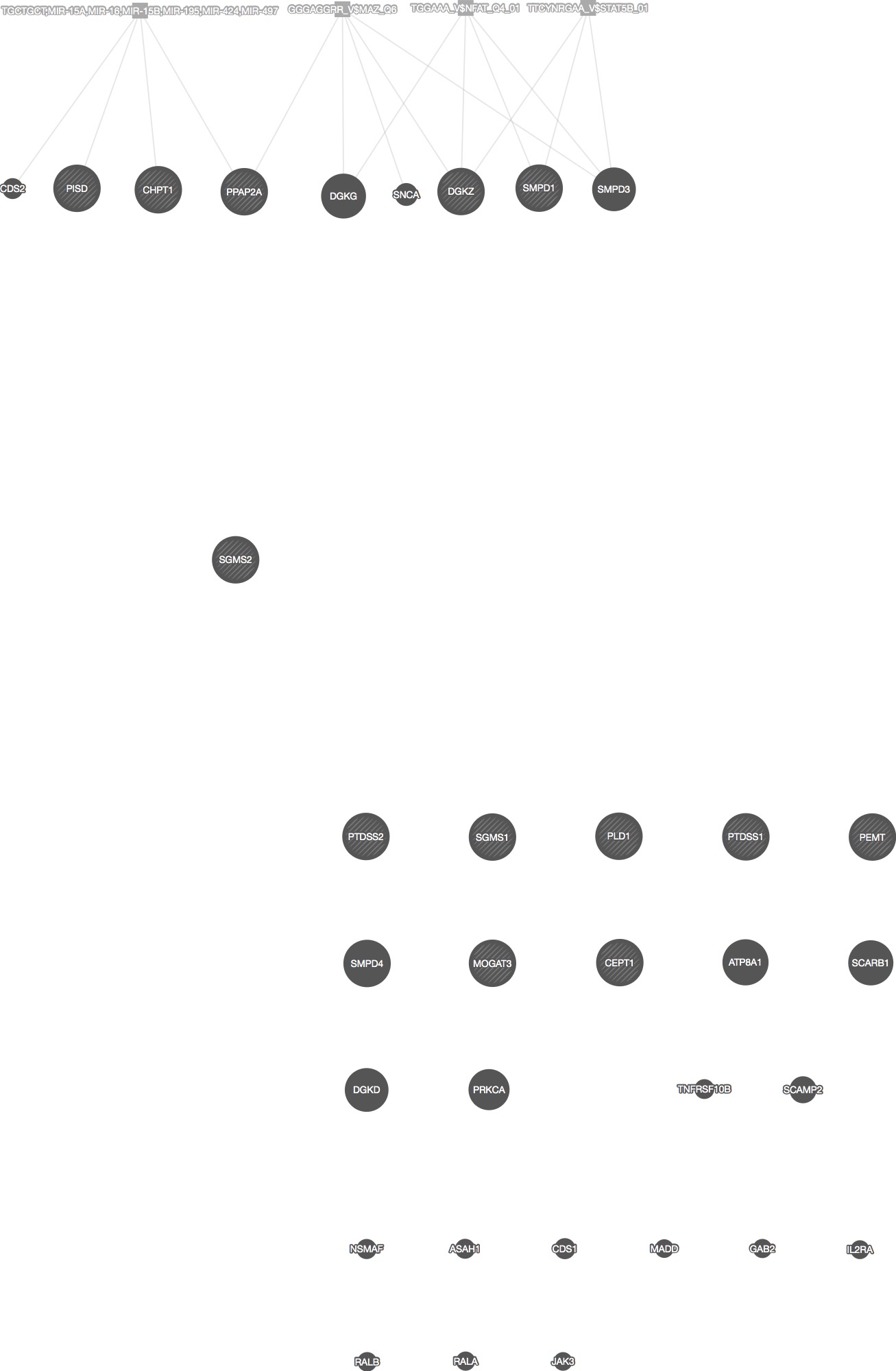
**

FigS10. First gene-regulatory and miRNA neighbours from GeneMania of the PCOS Important enzymes.

9. Advanced techniques

There several further ways in which an experienced bioinformatician can use the holistic lipidomic network generated here. The first would entail merging this network with others, such as BIOGRID, so that metabolic and regulatory pathways can be viewed simultaneously. Although a powerful approach, the resulting subnetworks can still be too large to interpret easily. A second way concerns looking beyond the immediate enzyme neighbours of perturbed metabolites to the next adjacent enzymes, as this enables potential synthesis and degradation pathways to be more clearly seen, but again the subnetworks become much larger. Where gene expression data (or other omics) are available, they can be used in a third way to colour code subnetworks to see if gene expression changes can account for the observed lipid profile changes.

References

Abdelmohsen et al. 2009 Ubiquitin-mediated proteolysis of HuR by heat shock. PMC2683047

Haoula, Z., S. Ravipati, D. Stekel, C. Ortori, C. Hodgman, C. Daykin, et al. (2015). Lipidomic analysis of plasma samples from women with polycystic ovary syndrome. Metabolomics 11, 657-666

Havugimana et al. 2012 A census of human soluble protein complexes. PMC3477804
