# Supplementary figures and images for: A methodology for elucidating regulatory mechanisms leading to changes in lipid profiles
Source: Metabolomics. 2017 May 29;13(7):81. doi: 10.1007/s11306-017-1214-y (PMC5447331; doi:10.1007/s11306-017-1214-y)

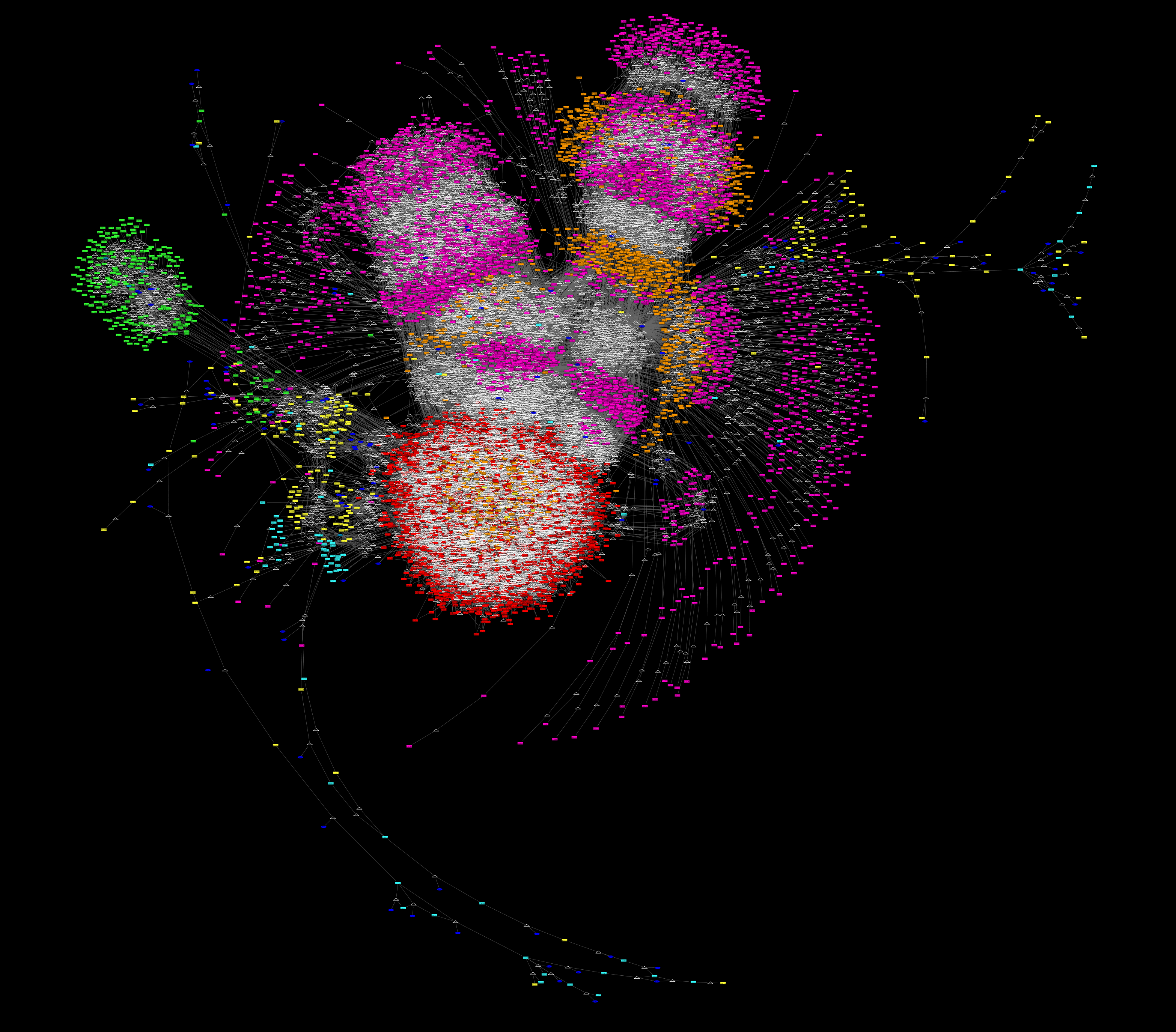

Supplement: Supplementary file 2 — Supplementary material 2 (TIFF 3845 KB) [file 11306_2017_1214_MOESM2_ESM.tiff]
